# Polyacrylic Acid/Polyaniline-Coated Multimode Interferometer for Ammonia Detection

**DOI:** 10.3390/ma16041478

**Published:** 2023-02-09

**Authors:** Ning Wang, Chao Zhao, Gang Long, Binyun Xia, Liang Wan, Kunpeng Niu, Jianguo Hou, Jiale Wang, Lei Lei, Zhichao Wang

**Affiliations:** 1 National Engineering Research Center of Fiber Optic Sensing Technology and Networks, Wuhan University of Technology, Wuhan 430070, China; 2 Zhongshan Institute of Modern Industrial Technology of SCUT, Zhongshan 528437, China; 3 Wuhan Bureau of Naval Equipment Department, Wuhan 430070, China

**Keywords:** coaxial optical fiber interferometer (COFI), polyacrylic acid, polyaniline, ammonia sensor

## Abstract

A coaxial optical fiber interferometer (COFI) is proposed here for ammonia sensing, which comprises two light-carrying single-mode fibers (SMF) fused to a section of no-core fiber (NCF), thus forming an optical interferometer. The outer surface of the COFI is coated with a layer of polyacrylic acid (PAA)/polyaniline (PAni) film. The refractive index (RI) of the sensitive layer varies when PAA/PAni interacts with ammonia, which leads to the resonance wavelength shift. The surface morphology and structure of the PAA/PAni composites were characterized by using a scanning electron microscope (SEM) and Fourier-transform infrared (FTIR) spectroscopy. When the sensor was exposed to an ammonia atmosphere of different concentrations at room temperature, the sensing performance of the PAA/PAni composite film was superior to that of a sensitive film formed by single-component PAA or PAni. According to the experimental results, the composite film formed by 5 wt% PAA mixed with 2 wt% PAni shows better performance when used for ammonia sensing. A maximum sensitivity of 9.8 pm/ppm was obtained under the ammonia concentration of 50 ppm. In addition, the sensor shows good performance in response time (100 s) and recovery time (180 s) and has good stability and selectivity. The proposed optical fiber ammonia sensor is adapted to monitor leakage in its production, storage, transportation, and application.

## 1. Introduction

Ammonia is widely used in the industrial production of fertilizers [1], plastics [2], explosives [3], etc. Ammonia leakage can not only pollute the environment but also cause potential security problems, especially for ammonia of high concentration. It has been reported that acute poisoning or life-threatening situations can occur after exposure to approximately 50 ppm of ammonia in the air [4]. Therefore, it is urgent to develop ammonia sensors with rapid response, high sensitivity, high accuracy, and a wide measurement range.

Various types of NH_3_ sensors have been reported during the last decades [5,6,7]. Among them, due to the merits of small size, resistance to electromagnetic interference, and high sensitivity [8], optical fiber sensors have been widely studied in the field of gas sensing [9,10,11,12]. For instance, Xu et al. [13] reported a highly sensitive NH_3_ sensor by coating graphene oxide (GO)/cellulose acetate (CA) on the surface of a long-period fiber grating (LPFG). Fu et al. [14] proposed an effective and simple method for detecting NH_3_ by coating the surface of a tapered microfiber interferometer with Fe_2_O_3_. The Fe_2_O_3_ nanotubes were used as the sensitive layer to enhance the adsorption of NH_3_ molecules and the intensity distribution of the evanescent field of the sensor, achieving an extreme range of NH_3_ detection. Fan et al. [15] proposed an NH_3_ sensor based on the Mach–Zehnder interferometer (MZI). A linear response to NH_3_ in the range of 0–151 ppm was achieved by coating the sensor surface with graphene oxide (GO). However, complex manufacturing processes or certain performance deficiencies prevented their practical application.

When an optical fiber sensor coated with a sensitive material is used for gas sensing, the gas will interact with the sensitive layer. This causes a change in the physical/chemical properties of the sensitive layer [16]. The layer’s optical properties (refractive index (RI), absorbance, etc.) are also changed. Currently, various materials have been applied for ammonia sensing, such as graphene oxide (GO) [15], graphene [17], metal oxides [18], PAni, etc. GO has a large specific surface area and good gas adsorption properties. However, GO also has poor film formation properties, which is not conducive to sensor applications. Graphene also has a large specific surface area and the ability to adsorb gases. Metal oxides also have their shortcomings. For example, gas sensors based on SnO_2_ have a low response at room temperature. PAni is widely used for NH_3_ sensing as its optical properties are altered in the presence of NH_3_. In the presence of NH_3_, PAni is reversibly deprotonated, changing from ES (emeraldine salt) to EB (emeraldine base) and back again after the NH_3_ has disappeared [19]. PAA is a weakly anionic polyelectrolyte that interacts strongly with alkaline gases such as NH_3_ [20]. The addition of water-insoluble PAni to PAA creates a porous sensitive film on the surface of the optical fiber sensor. This facilitates the entry and exit of gas molecules and further enhances the performance of the sensor. This work presents a novel coaxial optical fiber interferometer (COFI) for ammonia sensing, which is formed by fusing two pieces of single-mode fiber (SMF) with a section of no-core fiber (NCF) coated with the PAA/PAni composite film. Here, PAA enables PAni to form a homogeneous and continuous coating on the surface of the optical fiber and provides more adsorption sites for NH_3_ molecules. In addition, the performance of the sensor is further enhanced by varying the levels of polyaniline. The proposed sensor thus exhibits excellent sensitivity, response time, and selectivity for atmospheric environmental detection.

## 2. Experimental Section

### 2.1. Materials

Aniline (C_6_H_7_N, 99.5%), ammonium persulphate (APS, 98%), PAA (Mw = 5000, 50 wt% solution in water), sodium hydroxide (NaOH, 96%), hydrochloric acid (HCl, 36%), and ammonia solution (NH_3_•H_2_O) were purchased from Sinopharm Chemical Reagent Co., Ltd. (Shanghai, China). All the reagents were used without any further purification. The no-core fiber (OD: 125 µm) and SMF (G. 652) were purchased from Yangtze Optical Fiber and Cable Co., Ltd. (Wuhan, China).

The PAni powder was first synthesized by the typical chemical oxidative polymerization method [21]. During the synthesis processes, 10 mL of 0.4 M HCl solution was prepared and divided into two equal volumes. Subsequently, 0.2 g of aniline and 0.189 g of APS were respectively added into the HCl solutions and thoroughly stirred to mix them uniformly. After that, the two solutions were mixed and placed in a refrigerator (BCD-196DK, HOMA Appliances Co., Ltd. Zhongshan, China) at 0 °C for 30 min and kept there at 4 °C overnight. Finally, the reaction solution was taken out and centrifuged. The precipitate was then dried in a vacuum oven (DZF-6050ABF, Tianjin Gongxing Laboratory Instrument Co., Ltd., Tianjin, China) at 80 °C overnight, and the PAni powder was obtained.

### 2.2. Fabrication of the NH_3_-Sensing Probe

**Figure 1:**
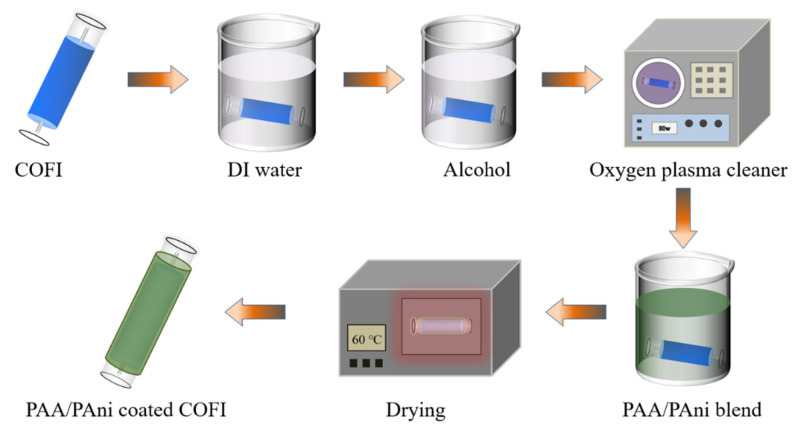
Process for the fabrication of sensing probes.

To fabricate the NH_3_-sensing probe, the COFI was made by fusing two sections of SMF to the ends of a section of NCF of about 3 cm using a fusion splicer (FSM-100P, Fujikura, Suzhou Laseropt Photonics Co., Ltd. Suzhou, China). The PAA/PAni mixtures were then prepared by adding different masses of PAni powder and PAA to DI water (deionized water). The content of PAni in the mixtures was 1 wt%, 2 wt%, 4 wt%, and 8 wt%, respectively, while the content of PAA remained constant at 5 wt%. Increasing the weight percentage of PAA in the mixture will result in a higher number of absorption sites, but again there is the problem of slow desorption. Therefore, the content of PAA was chosen to be 5 wt% in the experiments [20]. To obtain homogeneous mixtures, the suspension was sonicated for 24 h and then magnetically stirred overnight. Next, the NH_3_-sensing probes were prepared by coating a PAA/PAni composite film on NCF using a typical dip-coating method. The coating process for sensitive films is shown in Figure 1. Before coating, the surface of COFI was washed with DI water and alcohol. Then, the treatment was carried out using the oxygen plasma cleaner (power: 80 w; time: 300 s). The COFI was immersed in a PAA/PAni mixture for 3 h. It was then dried in an oven at 60 °C for 12 h.

### 2.3. Characterization of Sensitive Films on Sensing Probes

The sensitive films were characterized by microscope (Mako G-192B PoE, Statroda, Germany) and scanning electron microscopy (SEM) (Zeiss Merlin Compact, Oberkochen, Germany). The PAni, PAA, and PAA/PAni−2% were characterized by Fourier-transform infrared (FTIR) spectroscopy (Thermo Scientific Nicolet 6700 spectrophotometer, Waltham, MA, USA).

### 2.4. Working Principle

**Figure 2:**
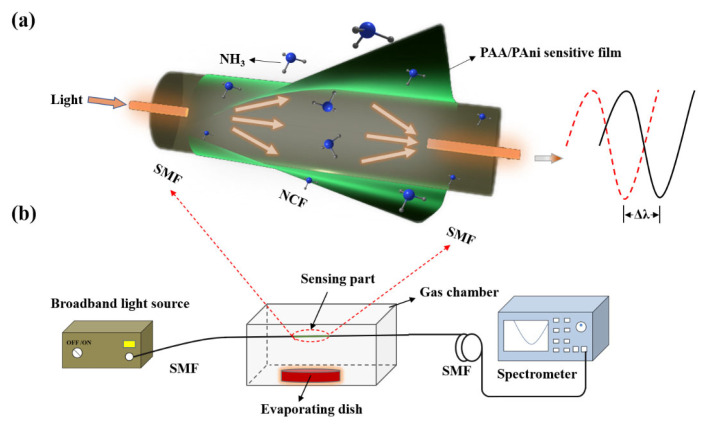
The optical fiber NH_3_ sensor coated with the sensitive PAA/PAni film. (**a**) Schematic diagram of the NH_3_-sensing probe; the green layer is the PAA/PAni composite film. (**b**) Experimental setup for NH_3_ detection.

Figure 2a shows the schematic diagram of the optical fiber NH_3_ sensor with SMFs fused to the ends of the NCF, which forms the optical fiber RI sensor. The green layer representing the PAA/PAni composite film is coated onto the COFI’s surface. When the light transports into the NCF from the SMF, multiple modes will be excited, resulting in multimode interference [21]. The mth-order interference peak wavelength can be expressed by

(1) $$\lambda_{m}=\frac{2\Delta n_{eff}L}{2m+1}\;$$

where $\Delta n_{eff}$ is the effective RI difference between the fundamental and higher-order modes. L is the length of the NCF. When the sensor is exposed to an ammonia atmosphere, the sensitive film on its surface will interact with the NH_3_. This will result in a variation in its effective RI and the resonance wavelength shift. Thus, it could be used for detecting NH_3_.

As shown in Figure 2b, the sensor is placed in the middle of the gas-tight chamber (volume ~ 7 dm^3^) with the chamber top cover for easily adjusting the NH_3_ concentration or filling the air. During the experiment, the gas chamber was placed in a fume hood. The SMFs were connected to the optical spectrum analyzer (OSA, AQ6370B, Yokogawa, Tokyo, Japan) and broadband light source (BBS, 1030–1660 nm), respectively. An evaporating dish heated to 250 °C was placed in the gas chamber for rapid ammonia evaporation to create the desired concentration of NH_3_ environment. The response of the optical fiber sensor at a certain NH_3_ concentration was recorded when the NH_3_ had diffused uniformly, and the stable spectrum was obtained. The experiment was carried out at room temperature, ignoring the influence of other potential external factors, and the pressure in the gas chamber was supposed to be the same as the atmospheric pressure.

## 3. Results

### 3.1. FTIR Analysis and Morphology of Composite Films

**Figure 3:**
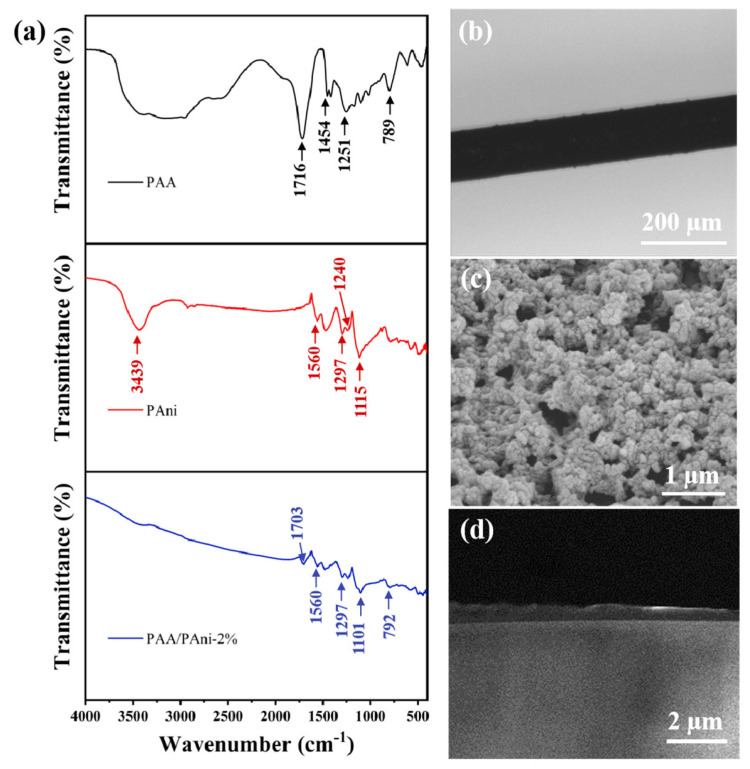
Characterization of NH_3_-sensitive films. (**a**) FTIR spectrum of the PAA, PAni, and PAA/PAni—2%. (**b**) Microscopic image of PAA/PAni—2% composite film coated on NCF. (**c**) The scaled-up image of PAA/PAni—2% composite film coated. (**d**) The cross-section of the probe coated with PAA/PAni—2%.

Figure 3a plots the FTIR spectra of the composite film (PAA/PAni—2%), PAA, and PAni. PAA presented stretching vibration bands of carbonyl groups at 1716 cm^−1^ [22]. The characteristic peak of PAni appears at near 1297 cm^−1^, which is attributed to the C–N stretching of the secondary aromatic amine [23]. In addition, the characteristic peaks approximately around 1560 and 1240 cm^−1^ come from the stretching of quinoid and the C–N^+^ stretching vibration [24], respectively. This indicates that PAni is successfully synthesized. The characteristic peak of PAA in the composite film appears at 1703 cm^−1^, which is attributed to the uncharged carboxylic group [25]. The characteristic peak of PAni (1297, 1560 cm^−1^) could still be found in the composite film. The above results indicate that the PAA/PAni—2% composite film has been successfully attached to the surface of the optical fiber. The microscopic image of PAA/PAni—2% composite film is exhibited in Figure 3b. The uniform and smooth film were also observed on the fiber. Figure 3c presents the SEM image of the PAni/PAA—2% composite film. The composite film shows a porous structure, which facilitates the entry and release of gas molecules. Figure 3d shows the cross-section of the probe coated with PAA/PAni—2%, and its thickness is approximately 400 nm.

### 3.2. Effect of Different Sensitive Films on Sensors

**Figure 4:**
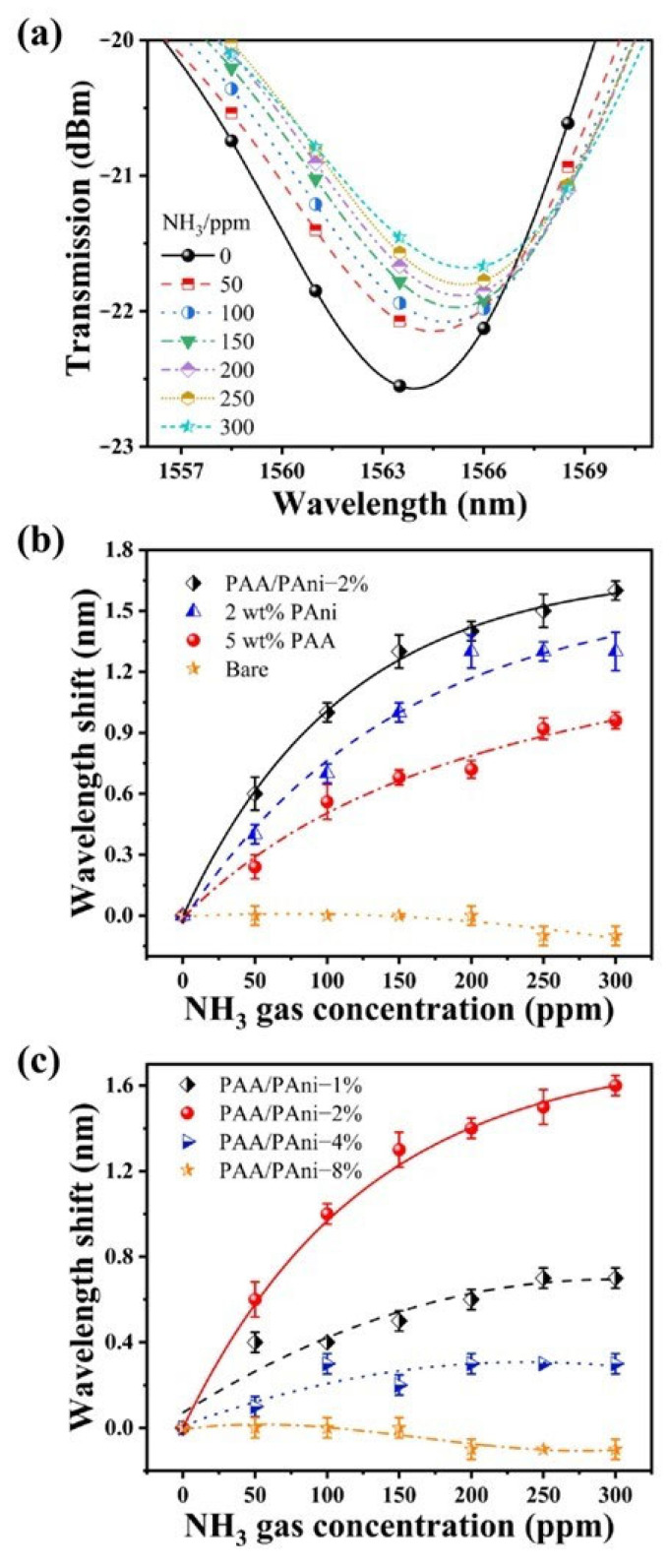
Effect of different sensitive films on the sensor’s response to NH_3_. (**a**)Transmission spectra of COFI sensor coated with PAA/PAni—2% under different NH_3_ concentrations. (**b**) Wavelength shift versus NH_3_ concentration when coated with different sensitive films. (**c**) The NH_3_ sensor’s performance when doping PAni of different content in PAA (5 wt%).

Figure 4a shows the transmission spectrum of the sensor at different NH_3_ concentrations. As the NH_3_ concentration changes from 0 to 300 ppm, the interference peak shifts from 1563.9 nm to 1565.5 nm. The effect of different sensitive films on the performance of the sensing probe is investigated, as shown in Figure 4b. According to the results, as the NH_3_ concentration increases, the resonance wavelength of the bare COFI sensor changes by only 0.1 nm, which could be regarded as the spectral fluctuations caused by the fluctuation of environmental factors. Thus, the bare COFI sensor did not respond to NH_3_. When the sensor was coated with PAA/PAni−2%, 2 wt% PAni, and 5 wt% PAA, respectively, the spectra were all red-shifted, which was related to the nature of the sensitive material. PAA is a weak anionic polyelectrolyte that interacts strongly with alkaline gas molecules such as NH_3_. At the same time, the ES form of PAni will be deprotonated or de-doped to the EB form of PAni when exposed to NH_3_. This causes the interference peak to be red-shifted. At different NH_3_ concentrations, optical fibers coated with composite films (PAA/PAni−2%) are better than single sensitive films (2 wt% PAni, 5 wt% PAA). The sensitivity can therefore be defined as 9.8 pm/ppm at the NH_3_ concentration of 50 ppm. To assess the effect of PAni content on sensor performance, the mass percentage of PAA was kept constant (5 wt%), and the mass content of PAni was varied (1 wt%, 2 wt%, 4 wt%, 8 wt%). As seen from the results in Figure 4c, the optimal response to NH_3_ of the sensor was obtained when the PAni content was kept at 2 wt%. The sensor performance was poor at the PAni content of 1 wt%. This is probably because the low content of PAni causes a nonuniform coating on the COFI. When the PAni content was controlled to be 4 wt% and 8 wt%, the poor sensor performance may be caused by the thick sensitive film with fewer pores blocking the gas molecules’ diffusion. The error bars here were acquired from three repeated experiments. Therefore, the composite sensitive film with the PAni content of 2 wt% (PAA/PAni—2%) was used when studying the other properties of the sensor (response time, stability, selectivity).

### 3.3. Response Time

**Figure 5:**
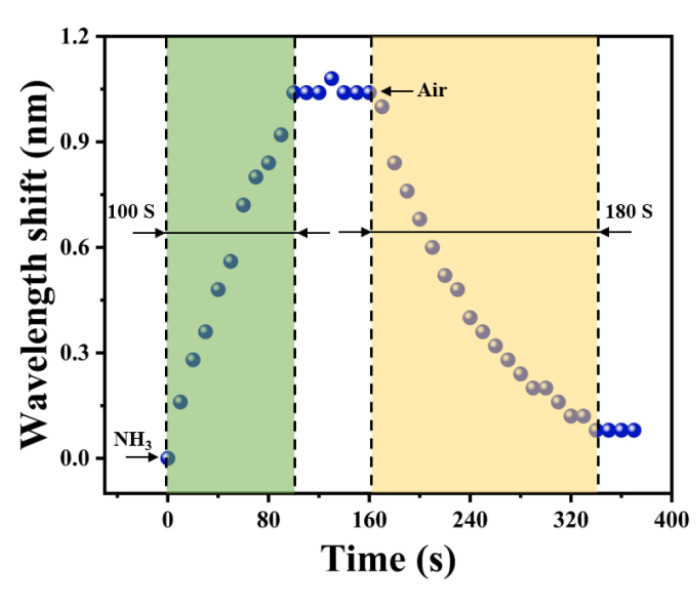
Response/Recovery time of the NH_3_ sensor.

Response time is one of the important indexes for evaluating the performance of gas sensors, and thus it is necessary to measure it to assess the feasibility of the sensor when used for practical detection. The response time of the sensor is read by automatically scanning the variation in the transmission spectrum continuously by the spectrometer. In the experiment, the data points in Figure 5 were recorded at 10 s intervals. When the sensor was placed in the 150 ppm ammonia atmosphere, it took only 100 s for the spectrum to stabilize. Subsequently, the gas chamber was opened and allowed to be filled rapidly with air. The recovery time of the sensor was recorded to be about 180 s.

### 3.4. Stability

**Figure 6:**
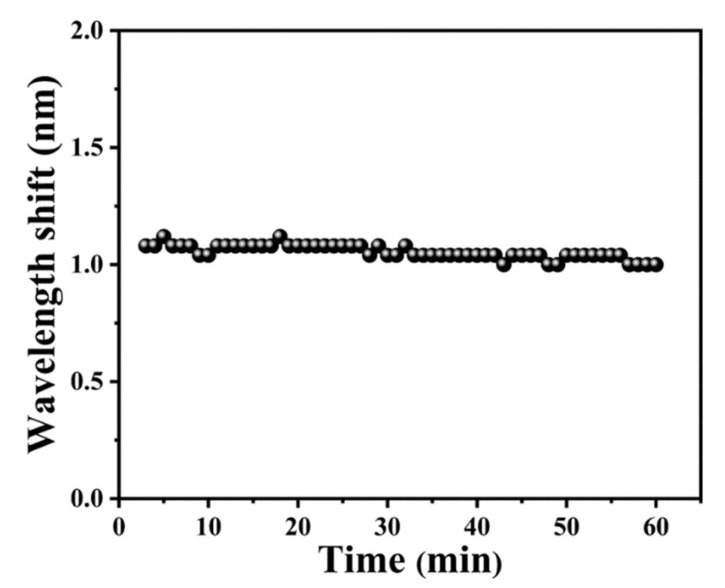
The stability of sensing probes.

The stability of the NH_3_ sensor was also investigated when controlling the NH_3_ concentration in the chamber at about 150 ppm. The NH_3_ sensor transmission spectra were recorded at 1 min intervals for 60 min. As shown in Figure 6, the maximum wavelength fluctuation of the sensor at the same NH_3_ concentration was about 0.18 nm, which is very small and could be ignored. Therefore, the probe exhibits good stability.

### 3.5. Selectivity of the NH_3_ Sensor

**Figure 7:**
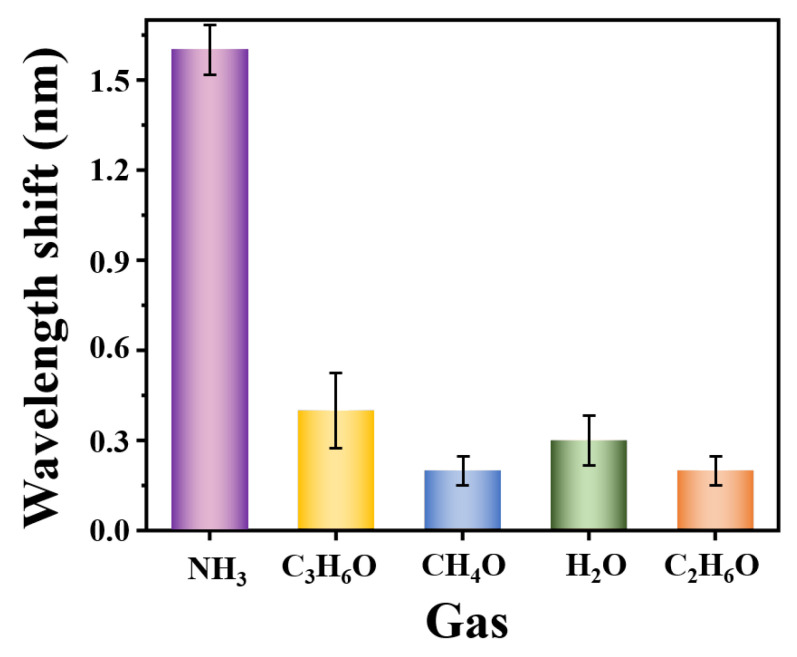
Response of the proposed sensor to different gases.

The possible presence of multiple gases in the real environment necessitates the study of the sensor’s selectivity. It was investigated at 25 °C when exposed to different target gases (NH_3_, C_3_H_6_O, CH_4_O, H_2_O, C_2_H_6_O) [14,18] at the constant concentration of 300 ppm. As shown in Figure 7, the wavelength shift of the sensor to NH_3_ is about 1.6 nm, while those to C_3_H_6_O, CH_4_O, H_2_O, and C_2_H_6_O are 0.4 nm, 0.2 nm, 0.3 nm, and 0.2 nm, respectively. Thus, the sensing probe exhibited excellent selectivity for NH_3_ compared to other interfering gases.

## 4. Discussion

Some of the currently reported optical ammonia sensors are listed in Table 1. Compared to previous studies, the PAA/PAni-based ammonia sensor proposed by us presents great performance, such as a wide detection range, high sensitivity, and fast response. The GO/CA-coated LPFG sensor [13] is the typical optical fiber NH_3_ sensor which is worthy of mention. In fact, when selecting gas sensors for practical applications, their comprehensive performance should be considered. The excellent sensitivity and response of the GO/CA-coated LPFG sensor have been demonstrated, which are better than most reported optical fiber ammonia sensors. However, its detection range obtained is 0–8.8 ppm, and thus, it could be developed for measuring the trace amount of NH_3_ in some specific fields, such as rapid disease diagnosis. The Fe_2_O_3_-coated tapered microfiber interferometer (MFI) [14] has a very wide detection range, but its sensitivity is correspondingly low. The GO-coated MZI sensor [15] also presents a wide detection range; however, its response time is too long to limit its practical application. The graphene-coated MZI sensor [17] is made of a pair of 3 dB LPFGs, which achieved good response in the NH_3_ concentration range of 10–180 ppm, while its response time is also long, and its fabrication is very complex. The graphene/microfiber hybrid waveguide (GMHW) sensor [26] has a wide detection range, but its preparation is too complex for its practical detection. The Pt/GO-coated microfiber sensor [27] demonstrated higher sensitivity but with a smaller detection range. The graphene-coated microfiber Bragg grating (GMFBG) sensor [28] is fabricated by using hydrofluoric acid etching. The response time/recovery time of the sensor is too long. The response/recovery time of the SnO_2_ sensor [29] is 175 s/210 s, respectively, when the ammonia concentration is kept at 50 ppm. It is obvious that the response time/recovery time of the sensor are both too long. Although the response time for the nanostructured Sb-doped SnO_2_ sensor [30] is shorter, the detection upper limit is very small to be 50 ppm. The metal carbide/carbonitride (MXene)/TiO_2_ sensor [31] is a self-powered device. This sensor has a response time/recovery time of 76 s/62 s. The concentration measuring range of this sensor is slightly inadequate compared to our proposed sensor. Currently, some commercial ammonia-sensing probes have the disadvantage of a small gas detection range, such as the ME3-NH_3_ ammonia sensor and ME4-NH_3_ ammonia sensor from Winsen technologies [32]. The low sensitivity of our proposed sensor is largely limited by the optical fiber structure, which can be improved by using an inherently more sensitive fiber structure as a substrate, such as an excessively tilted fiber grating. The problem of long response times could also be promoted by optimizing the sensitive film porous structure and coating technique which might bring more active sites and pathways for the interaction between the gas molecules and sensitive materials. Although the optical fiber ammonia sensors reported above have their own advantages and limitations, the detection performance, applicability, practicability, and cost must be considered in consideration of practical applications.

## 5. Conclusions

In summary, a COFI-based ammonia sensor with high sensitivity and fast response has been developed here. By employing the PAA/PAni composite materials as a sensitive layer, the optical fiber NH_3_ sensor has been demonstrated with great performance. It was found that the sensitivity of the sensor coated with the PAA/PAni composite film was more sensitive than that of the sensor coated with a single sensitive film (PAA or PAni). In addition, the performance of the sensing probe also depends absolutely on the doping amount of PAni in the composite film, while the optimal performance was explored when the PAni content was adjusted to be ~2 wt%. The sensor showed a good response over a wide detection range of ammonia concentrations from 0 to 300 ppm. The detection sensitivity was obtained to be 9.8 pm/ppm at the NH_3_ concentration of 50 ppm. The response and recovery times of the sensor were 100 s and 180 s, respectively. In addition, the proposed sensor exhibited good stability and specificity. Obviously, the optical fiber ammonia sensor developed by us is still in the laboratory stage. However, it has the potential to enable the low-cost optical fiber ammonia sensor with excellent performance that can be utilized easily and effectively in practical applications.

## Figures and Tables

**Table 1:** Comparison of different NH_3_ sensors.

NH_3_ Sensors	Detection Range (ppm)	Sensitivity	Response/Recovery Time	Ref.
GO/CA-coated LPFG	0–8.8	98.32 pm/ppm	32 s/68 s	[13]
Fe_2_O_3_-coated MFI	0–11,640	1.30 pm/ppm	/	[14]
GO-coated MZI	0–151	4.97 pm/ppm	5 min/7.5 min	[15]
Graphene-coated MZI	10–180	3 pm/ppm	270 s/-	[17]
GMHW	40–360	6 pm/ppm	0.5 s/-	[26]
Pt/GO-coated microfiber	0–120	10.2 pm/ppm	/	[27]
GMFBG	0–100	4 pm/ppm	~10 min/~15 min	[28]
SnO_2_	50	694.4%	175 s/210 s	[29]
Nanostructured Sb-doped SnO_2_	50	4316%	70 s/-	[30]
MXene/TiO_2_	1–100	6.84% (10 ppm)	76 s/62 s	[31]
ME4	0–50	(0.12 ± 0.03) uA/ppm	≤90 s/-	[32]
PAA/PAni-coated COFI	0–300	9.8 pm/ppm	100 s/180 s	Our work

## Data Availability

Not applicable.
